# Assessment of Neurovascular Involvement by Malignant Musculoskeletal Tumors

**DOI:** 10.1080/13577149778506

**Published:** 1997-03

**Authors:** David M. Panicek, Susan Hilton, Lawrence H. Schwartz

**Affiliations:** Department of Radiology Memorial Sloan-Kettering Cancer Center 1275 York Avenue New York NY 10021 USA

## Abstract

Determining the presence or absence of neurovascular involvement by a malignant musculoskeletal neoplasm is an important aspect of local tumor staging. This article discusses issues concerning such assessments made by diagnostic imaging techniques, including factors inherent to the patient and those related to imaging technology. The distinction between tumor contact and tumor encasement is emphasized and illustrated.


*Sarcoma* (1997) **1**, 61–63

ISSUES IN IMAGING

### Assessment of neurovascular involvement by malignant musculoskeletal tumors

DAVID M. PANICEK, SUSAN HILTON & LAWRENCE H. SCHWARTZ

*Department of Radiology, Memorial Sloan-Kettering Cancer Center, New York, USA*

**Abstract**
Determining the presence or absence of neurovascular involvement by a malignant musculoskeletal neoplasm is an important aspect of local tumor staging. This article discusses issues concerning such assessments made by diagnostic imaging techniques, including factors inherent to the patient and those related to imaging technology. The distinction between tumor contact and tumor encasement is emphasized and illustrated.

**Key words:** *sarcoma, staging, blood vessel, nerve, invasion, computed tomography, magnetic resonance imaging.*

One of the major contributions of imaging to the management of a patient with sarcoma is in defining the local extent of the tumor. By providing the team of treating physicians with an accurate assessment of the various osseous, articular, muscular and neurovascular structures that are contacted or invaded by tumor, the radiologist can help to maximize the success of subsequent interventions while minimizing the amount of tissue (and function) that is removed.[1–4]

The presence of neurovascular invasion can have therapeutic and prognostic implications. Unfortunately, several factors limit the radiologic assessment of neurovascular structures:

(1) Arteries, veins and especially nerves are small structures, placing great demands on imaging studies to depict the structures and their relationship to a tumor.
(2) Most peripheral nerves tend to 'blend in' visually with adjacent muscles on CT and magnetic resonance imaging (MRI). Only the larger peripheral nerves are generally identifiable as discrete structures on these imaging studies; the location of other nerves is generally inferred based on their expected location and relationship to adjacent structures.
(3) The identification of nerves is further limited when normal anatomic relationships are distorted by the presence of a tumor mass.
(4) It can be difficult or impossible to differentiate edema in the reactive zone surrounding a tumor and the tumor itself at imaging,[5–7] limiting the evaluation of the neurovascular structures present within that abnormal tissue.
(5) Non-invasive cross-sectional imaging techniques such as CT and MRI can demonstrate whether a tumor is close to or in contact with a neurovascular structure (Fig. 1), but usually cannot differentiate mere contact, adherence or subtle invasion (Fig. 2). Gross encasement of a vessel can be diagnosed reliably only when a tumor mass clearly surrounds the vessel (Fig. 3). Irregularity of vessel walls shown at angiography can be due to tumor encasement or atherosclerosis.
(6) The prevalence of neurovascular involvement by sarcomas is low, which has an important effect on the positive predictive values of imaging test results. For example, in the Radiology Diagnostic Oncology Group multi-institutional collaborative trial which compared CT and MRI in local staging of malignant musculoskeletal neoplasms, the prevalence of

Correspondence to: D. M. Panicek, Department of Radiology, Memorial Sloan-Kettering Cancer Center, 1275 York Avenue, New York, NY 10021, USA. Tel: +1 212 639 5825; Fax: +1 212 794 4010; E-mail: panicekd@mskcc.org.

1357-714X/97/010000-00 ©1997 Journals Oxford Ltd

(a) (b)

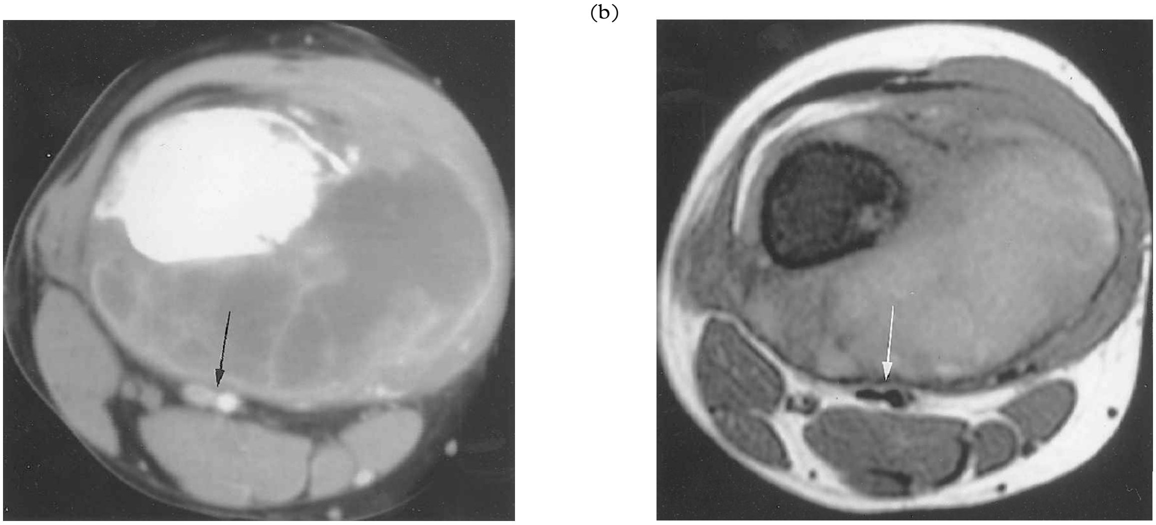

**Fig. 1.** *Vascular displacement, but no direct contact with tumor: (a) contrast-enhanced CT scan and (b) T1-weighted (600/12) MR image. The large soft tissue mass due to osteogenic sarcoma of the distal femur displaces the popliteal vessels posteriorly. The tissue plane visible between the vessels (arrow) and the margin of the mass indicates that no gross tumor involvement of the vessels has occurred.*

(a) (b)

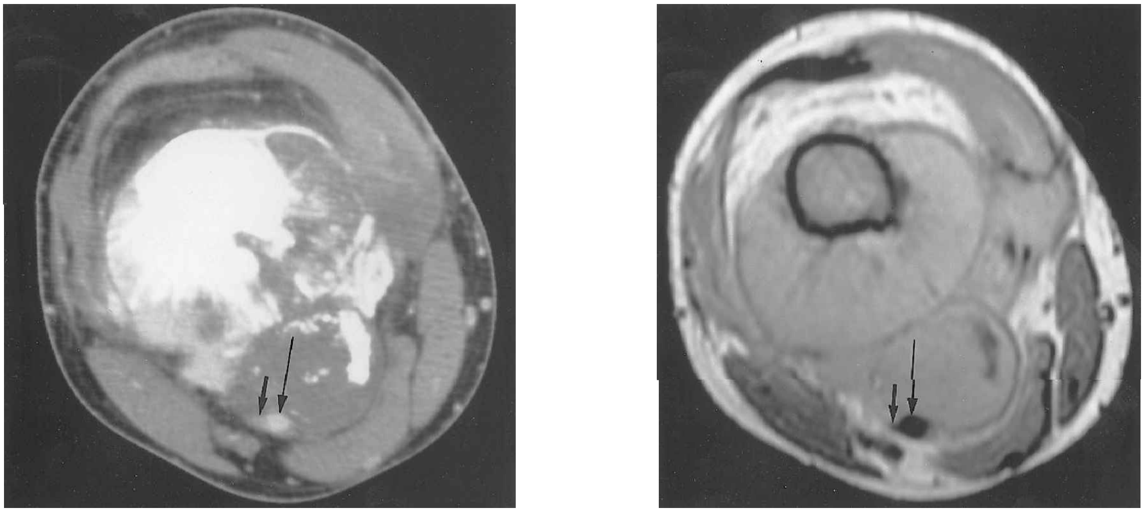

**Fig. 2.** *Vascular displacement and direct contact with tumor, but no encasement: (a) contrast-enhanced CT scan and (b) proton density-weighted (2466/20) MR image. The large soft tissue mass due to osteogenic sarcoma of the distal femur is in intimate contact with the popliteal artery (long arrow) and vein (short arrow); no tissue plane is evident between the mass and the vessels. Although both CT and MRI were interpreted as showing encasement of the vessels, none was present at surgery.*

vascular involvement was 3.3% and neural involvement was 1.1% for the 183 primary bone tumors studied; corresponding figures for the 133 primary soft tissue tumors were 4.5% and 6.8%, respectively.[8] The positive predictive value of CT and MRI for neurovascular involvement by sarcomas in that study was only 6–27%, with a negative predictive value of 92–99%.

In view of these limiting factors, what can be done to improve the assessment of neurovascular structures at imaging of sarcoma? The importance of excellent image quality is obvious. Large imaging matrices and small field-of-view imaging focused on the local tumor site are required, as is use of appropriate MRI surface coils. For CT, intravenous contrast material delivered by rapid bolus injection should be used to optimize the delineation of vessels. Special CT or MRI angiographic sequences may be of value, as well. When interpreting the images, it is advisable to remember the relatively low prevalence of neurovascular invasion and thus to limit the tendency to over-diagnose invasion based

(a) (b)

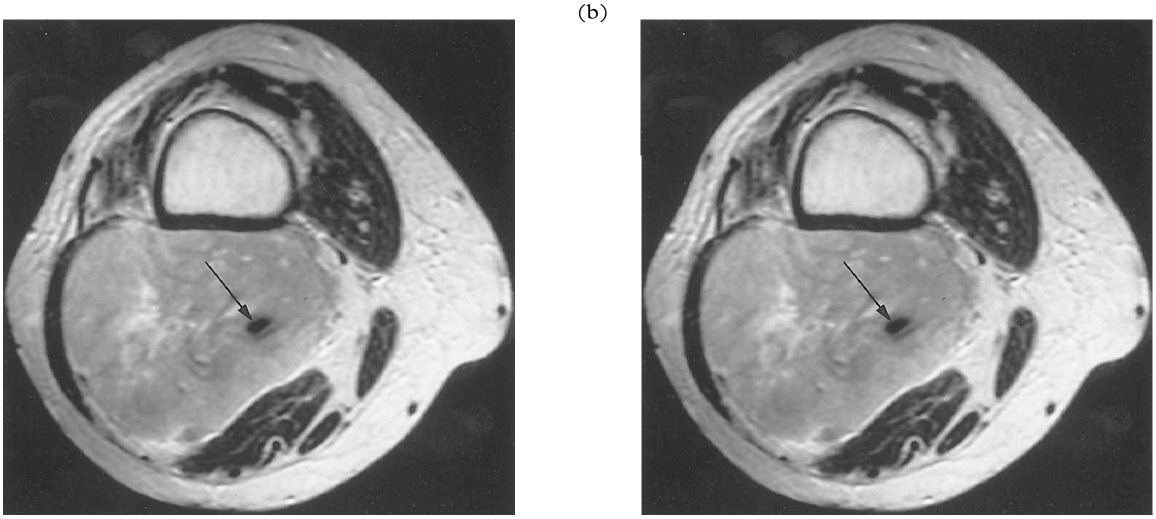

**Fig. 3.** *Gross encasement of vessels by tumor: (a) contrast-enhanced CT scan and (b) T2-weighted (2000/80) MR image. The popliteal artery (arrow) is clearly located deep within the large leiomyosarcoma of the distal thigh. The popliteal vein is not visible because it is encased and compressed by the mass.*

solely on the demonstration of contact between tumor and neurovascular structures.

### References

1 Enneking WF, Spanier SS, Goodman MA. The surgical staging of musculoskeletal sarcoma. *J Bone Joint Surg* 1980; 62A:1027–30.
2 Enneking WF, Spanier SS, Malawer MM. The effect of the anatomic setting on the results of surgical procedures for soft parts sarcoma of the thigh. *Cancer* 1981; 47:1005–22.
3 McDonald DJ. Limb-salvage surgery for treatment of sarcomas of the extremities. *AJR* 1994; 163:509–13.
4 Smith DK, Parsons TW. Re: limb-salvage surgery for treatment of sarcomas of the extremities. *AJR* 1994; 163:514–16.
5 Beltran J, Simon DC, Katz W, *et al.* Increased MR signal intensity in skeletal muscle adjacent to malignant tumors: pathologic correlation and clinical relevance. *Radiology* 1987; 162:251–5.
6 Seeger LL, Widoff BE, Bassett LW, *et al.* Preoperative evaluation of osteosarcoma: value of gadopentetate dimeglumine-enhanced MR imaging. *AJR* 1991; 157: 347–51.
7 Shuman WP, Patten RM, Baron RL, *et al.* Comparison of STIR and spin-echo MR imaging at 1.5 T in 45 suspected extremity tumors: lesion conspicuity and extent. *Radiology* 1991; 179:247–52.
8 Panicek DM, Gatsonis C, Rosenthal DI, *et al.* CT and MR imaging in the local staging of malignant musculoskeletal neoplasms: report of the Radiology Diagnostic Oncology Group. *Radiology* 1997; 202:237–46.
